# Protoporphyrins Enhance Oligomerization and Enzymatic Activity of HtrA1 Serine Protease

**DOI:** 10.1371/journal.pone.0115362

**Published:** 2014-12-15

**Authors:** Hakryul Jo, Victoria Patterson, Sean Stoessel, Chia-Yi Kuan, Josephine Hoh

**Affiliations:** 1 Department of Environmental Health Science, Yale University School of Public Health, New Haven, Connecticut, United States of America; 2 Department of Ophthalmology and Visual Sciences, Yale University School of Medicine, New Haven, Connecticut, United States of America; 3 Department of Pediatrics, Emory University School of Medicine and Children’s Healthcare of Atlanta, Atlanta, Georgia, United States of America; Saitama Medical University, Japan

## Abstract

High temperature requirement protein A1 (HtrA1), a secreted serine protease of the HtrA family, is associated with a multitude of human diseases. However, the exact functions of HtrA1 in these diseases remain poorly understood. We seek to unravel the mechanisms of HtrA1 by elucidating its interactions with chemical or biological modulators. To this end, we screened a small molecule library of 500 bioactive compounds to identify those that alter the formation of extracellular HtrA1 complexes in the cell culture medium. An initial characterization of two novel hits from this screen showed that protoporphyrin IX (PPP-IX), a precursor in the heme biosynthetic pathway, and its metalloporphyrin (MPP) derivatives fostered the oligomerization of HtrA1 by binding to the protease domain. As a result of the interaction with MPPs, the proteolytic activity of HtrA1 against Fibulin-5, a specific HtrA1 substrate in age-related macular degeneration (AMD), was increased. This physical interaction could be abolished by the missense mutations of HtrA1 found in patients with cerebral autosomal recessive arteriopathy with subcortical infarcts and leukoencephalopathy (CARASIL). Furthermore, knockdown of HtrA1 attenuated apoptosis induced by PPP-IX. These results suggest that PPP-IX, or its derivatives, and HtrA1 may function as co-factors whereby porphyrins enhance oligomerization and the protease activity of HtrA1, while active HtrA1 elevates the pro-apoptotic actions of porphyrin derivatives. Further analysis of this interplay may shed insights into the pathogenesis of diseases such as AMD, CARASIL and protoporphyria, as well as effective therapeutic development.

## Introduction

High temperature requirement protein A1 (HtrA1), a secreted serine protease of the HtrA family, has important functions in protein quality control and physiological processes [1], [2]. The HtrA1 protein is predominantly secreted to effect degradation of extracellular matrix proteins such as fibronectin and Fibulin-5, although a small amount is retained within the cell where it has roles in protein processing and degradation [3]–[6]. The protease function of HtrA1 is regulated by the formation of oligomers, with the trimer thought to be the basic catalytic unit [2], [7]–[9]. Unlike bacterial HtrA proteins, mammalian HtrA1 is likely regulated by substrate-induced remodeling of the active site rather than by the PDZ domain [10], [11].

Mutation or misregulation of HtrA1 is associated with diverse diseases in humans. HtrA1 hyperactivity contributes to arthritis, while single-nucleotide polymorphisms in the *HtrA1* promoter have been associated with increased risk of AMD [12]–[15]. Loss of protease activity, or a reduction in *HtrA1* expression, is associated with CARASIL and increased TGFβ signaling, as well as tumorigenesis and metastasis in several cancers [16]–[20]. Down regulation of HtrA1 is also associated with a resistance to chemotherapy–induced cytotoxicity [21].

Protoporphyrin IX (PPP-IX), a physiological precursor in the heme biosynthetic pathway, is used in photodynamic therapy for treating cancer: protoporphyrin absorbs light radiation and emits reactive oxygen species to damage cancerous cells [22]–[24]. PPP-IX accrues to toxic levels in Erythropoietic protoporphyria (EPP), resulting in acute photosensitivity of skin [25]. Protoporphyrin is also capable of triggering the mitochondrial permeability transition and apoptosis independently of photosensitization [26]. Whether protoporphyrin modulates the HtrA1 activity has not been demonstrated to this date.

The exact role of HtrA1 in cellular processes and disease progression is unclear. Here, we report the screening a library of small molecules for their ability to alter extracellular HtrA1 oligomer formation. We identified two metalloporphyrins (MPPs) among the positive hits and demonstrate that MPPs interact with the catalytic domain of HtrA1 and increase its enzymatic activity, as assessed by Fibulin-5 cleavage. Selected CARASIL-associated missense mutations abolished this interaction. Finally, we showed that knockdown of HtrA1 attenuates PPP-IX induced cell death. To our knowledge, the present study is the first report of a direct protoporphyrin-HtrA1 interaction, which sheds new insights into the pathological mechanisms of prophyrias and HtrA1-related disorders.

## Materials and Methods

### Reagents and antibodies

Metalloporphyrins and protoporphyrin IX (Santa Cruz biotechnology) were dissolved in DMSO and stored in the dark. Chemicals were purchased from Sigma Aldrich except HEMIN (MP Biomedicals), rosmarinic acid (Cayman Chemical) and CCCP (Santa Cruz Biotech). All antibodies were purchased from Cell Signaling Technology except monoclonal anti-HtrA1 and polyclonal anti-HtrA2 (R&D Systems), rabbit polyclonal anti-Fibulin-5 (Millipore), monoclonal anti-V5 (Invitrogen), and rabbit polyclonal anti-HtrA1 (kind gift from Dr. Sascha Fauser, University of Cologne [4]).

### Cell culture

HEK293 and HeLa cell lines were obtained from American Type Culture Collection (ATCC). Cells were maintained in standard media (10% fetal bovine serum, 1% penicillin and streptomycin in DMEM) under 5% CO_2_. Lipofectamine 2000 (Invitrogen) was used to transfect plasmids and siRNAs following the manufacturer’s instruction. Stably transfected cell lines were generated by transfecting wild type or variant human *HtrA1* constructs into HEK293 cells and selected for their resistance to G418 (2 mg/ml). Stably transfected cells were maintained in standard medium plus G418 (0.5 mg/ml). The human *HtrA1* siRNA (5′-GGUGAAGUGAUUGGAAUUATT-3′; 5′-UAAUUCCAAUCACUUCACCTT-3′) and corresponding negative control siRNA (5′-UUCUCCGAACGUGUCACGUTT-3′; 5′-ACGUGACACGUUCGGAGAATT-3′) were purchased from Abgent, Inc.

### Cloning

Standard molecular biological techniques were used for subcloning of *HtrA1*. An HA tag was inserted downstream of the signal peptide of human *HtrA1* cDNA (clone obtained from Origene, SC118403) and the construct was cloned into pCDNA3.1/V5-His-TOPO vector to generate a *HtrA1* expression vector. Site-directed mutagenesis of this contruct was performed to generate *HtrA1* mutations identified in CARASIL patients and standard PCR amplification was used to generate *HtrA1* deletion constructs. All constructs were confirmed by sequencing.

### Protein purification and separation

Ni-NTA columns (Qiagen) were loaded with HtrA1 conditioned medium containing 20 mM imidazole and 0.05% NP-40, washed three times (20 mM imidazole, 0.05% NP-40/PBS) and bound fractions were eluted (200 mM imidazole, 0.05% NP-40/PBS). Eluted fractions were subjected to centrifugal filter devices (Amicon) four times to remove excess imidazole and to concentrate the proteins (2000 g, 30 min, 4°C). Purified protein was reconstituted in PBS.

Samples were resolved on SDS-PAGE (4–20% gradient, Bio-rad) in the presence or absence of reducing agent (355 mM β-mercaptoethanol), transferred onto PVDF membrane (Bio-rad), blocked (5% non-fat dry milk/140 mM NaCl/10 mM Tris pH 8 (TBS-T)) and incubated in primary antibody (1∶1000 in 0.1% sodium azide/3% BSA/TBS-T) overnight (4°C). After washing, membranes were incubated in HRP-conjugated secondary antibodies (1∶5000 in 5% non-fat dry milk/TBS-T). Signal was detected using Western Lightning Plus (Perkin Elmer).

### Chemical intervention screen

HEK-HtrA1 and HeLa cells were cultured (standard medium, 48 hr) and conditioned medium was collected (18500 g, 10 min, 4°C) and diluted with serum free DMEM (1∶3). Individual chemicals from a library of bioactive compounds (Tocriscreen, http://www.tocris.com/screeningLibraries.php) described in previous screens [27]–[30] were added to conditioned medium to a final concentration of 25 mM. Reaction mixtures were incubated (1 hr, 37°C) and reaction was stopped by addition of 1x Laemmli Sample Buffer (Bio-rad, 50 µl). Samples were denatured (5 min, 95°C), subjected to non-reducing SDS-PAGE and analyzed by immunoblotting for HA. A DMSO control was included on every blot to allow comparison. The initial compounds of interest were identified as reproducibly affecting the level of monomeric HtrA1 or its complexes, compared to DMSO.

### MPP treatment and HEMIN-agarose affinity chromatography

To test the effect of MPPs on extracellular HtrA1 complex formation, cells were cultured (0.2% FBS/DMEM, 48 hours) then CM was collected and incubated with MPPs (25 mM, 1 hr, 37°C). Samples were denatured (5 min, 95°C) following addition of 1X Laemmli Sample Buffer.

For affinity chromatography, cells were lysed to obtain intracellular HtrA1 (1% NP-40/PBS plus protease inhibitor cocktail, 15 min, 4°C) and lysates centrifuged (18500 g, 10 min, 4°C). Protein lysate (1 mg/500 µl) or conditioned medium (500 µl, containing extracellular HtrA1) was incubated with HEMIN-agarose slurry (30 µl, overnight, 4°C), beads were washed three times (0.1% NP-40/PBS, 4°C) and bound proteins were eluted (50 µl 2x Laemmli Sample Buffer (Bio-rad)). For competitive binding experiments, free MPPs were pre-incubated with conditioned medium or protein lysate (1 hr, 4°C) prior to addition of HEMIN-agarose beads.

### Enzyme-linked Immunosorbent Assay

To prepare the ELISA plate, monoclonal anti-V5 antibody (100 ng/well) was incubated in a 96 well plate (Pierce Biosciences, overnight, 4°C). Unbound antibody was removed by extensive washing (0.05% NP-40/PBS). Purified HtrA1 protein (500 ng/100 µl) was mixed with individual chemicals and incubated with the ELISA plate (overnight, 4°C). After washing (0.05% NP-40/PBS), rabbit anti-HA antibody (100 ng/well) was added (2 hr, 4°C). The plate was washed (TBS-T) and HRP-conjugated rabbit secondary antibody (1∶1000) was added. Subsequent ELISA steps were performed using the femto-HRP ELISA kit (G biosciences) according to manufacturer’s instructions.

### Fibulin-5 degradation assay

Conditioned medium from HEK-HtrA1 cells was incubated (37°C) with methanol-fixed HeLa cells in the presence of metalloporphyrins or DMSO. The extracellular matrix fraction was then collected and analyzed by immunoblotting for Fibulin-5.

### Caspase-3 activity and cell viability assay

Cell viability was determined by the MTT assay as described previously [31]. Briefly, drug-treated HeLa cells were cultured (0.2% serum/DMEM minus phenol red), incubated with MTT solution (50 µl of 5 mg/ml in DMSO/PBS, 2 hr, 37°C) and crystals were dissolved by addition of 0.1 M HCl/isopropanol. Well contents were centrifuged (18500 g, 5 min, 4°C) and the absorbance of the supernatant was measured at 570 nm. The caspase 3/7 glo assay kit (Promega) was used to measure caspase-3 activity. HeLa cells transfected with siRNA (2×10^5^) were plated in triplicate (standard medium, 24 hr). Cells were incubated with individual MPPs then media was replaced (0.2% serum/DMEM). An equal volume of caspase 3/7 substrates was added and aliquots (200 µl) were transferred to a white-walled 96 well plate for luminometer reading.

## Results

### Secreted HtrA1 forms molecular complexes dependent on cell culture conditions

In order to screen for co-factors of HtrA1, we first assessed the culture conditions that influence the conformation of secreted HtrA1. To do so, we cultured HeLa cells in rich (10% FBS) or low (0.2% FBS) serum media for 24 to 72 hr, and the HtrA1-containing conditioned medium (CM) was analyzed by SDS-PAGE in the presence or absence of the reducing agent β-mercaptoethanol (β-ME).

**Figure 1:**
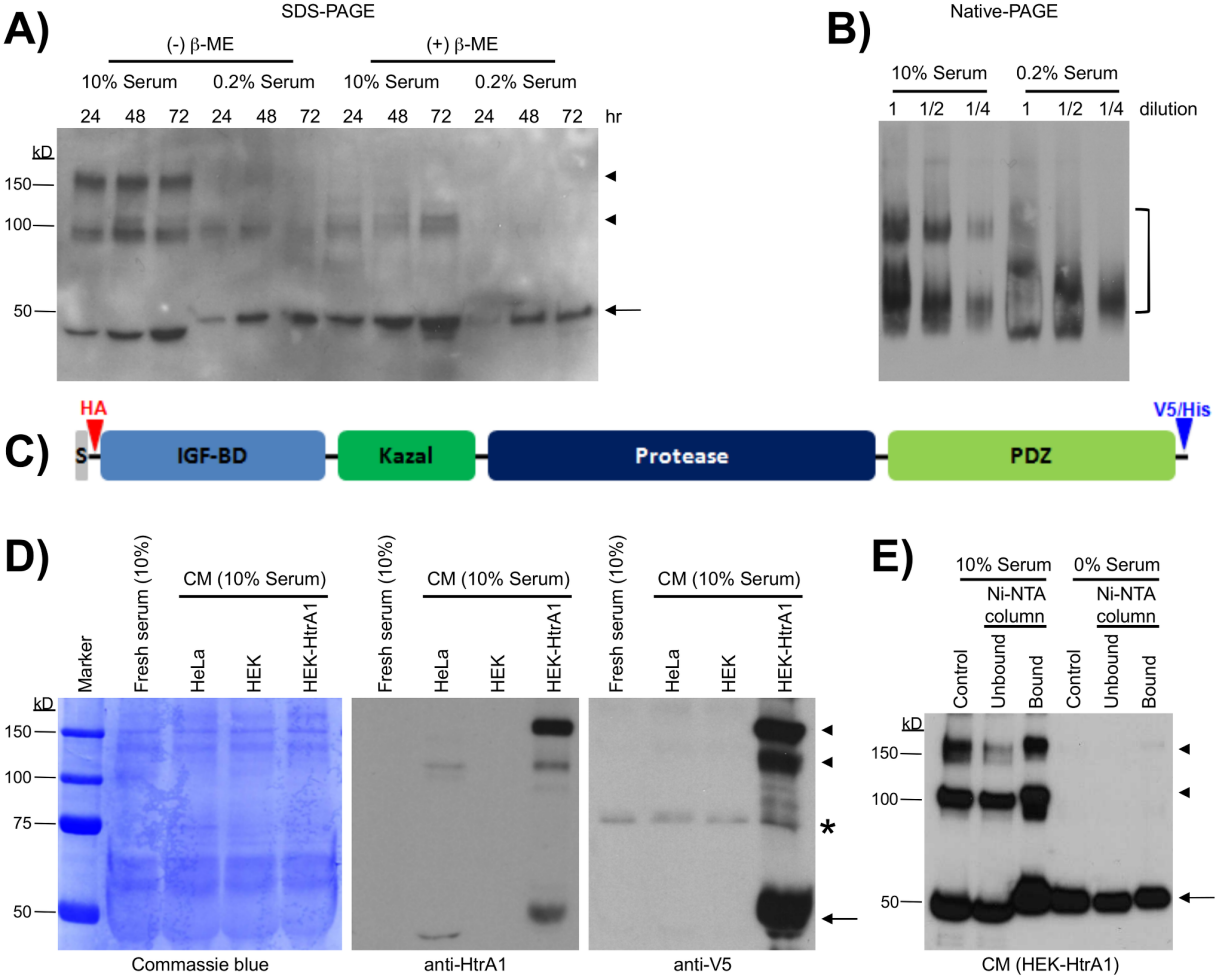
Formation of oligomer complexes by secreted HtrA1 protein in cell culture. (**A**) Serum rich (10%) conditioned medium from HeLa cells contained a higher level of HtrA1 complexes than low serum (0.2%) conditioned medium. Medium was collected at the indicated time points and subjected to non-reducing (- β-ME) or reducing (+ β-ME) SDS-PAGE. Immunoblotting with polyclonal anti-HtrA1 antibody detected full length HtrA1 (arrow) and HtrA1-containing complexes (arrowheads) under non-reducing conditions. Full length HtrA1 could be detected under non-reducing conditions. (**B**) Serum rich and low serum conditioned media subjected to native PAGE revealed two prominent bands when probed with monoclonal anti-HtrA1 antibody (bracket). A dilution series is shown for clarity. (**C**) A schematic diagram of the human HtrA1 expressed from the full-length construct, tagged with an HA epitope (red) at the N-terminus and V5/hexa-His tag (blue) at the C-terminus. S denotes the signal peptide. (**D**) Exogenous HtrA1 is stably expressed in HEK293 cells. HtrA1 could be detected in conditioned medium from HeLa cells and stably transfected HEK-HtrA1 cells, but not that from HEK293 cells or medium that has not been exposed to cells, when probed with monoclonal anti-HtrA1 antibody (middle panel). Probing with anti-V5 antibody (right panel) detected specific bands (arrows, arrowheads) only in HEK-HtrA1 conditioned medium, although a non-specific band was detected in all samples (asterisk). Coomassie blue staining is included as a loading control (left panel). (**E**) Serum rich (10%) conditioned medium contained a higher level of exogenous HtrA1 complexes than serum free conditioned medium. Exogenous HtrA1 was captured from conditioned medium via the hexa-His tag, using Ni-NTA columns. Full-length HtrA1 (arrow) could be detected in the input conditioned medium (control) and column-captured fraction (bound) under both serum conditions. HtrA1 complexes (arrowheads) were only detected under serum rich conditions. CM: conditioned medium.

Full-length, monomeric HtrA1 (∼50 kDa) was present at all time points in high-serum medium and, to a lesser degree, in low-serum culture (Fig. 1A). In addition to monomeric HtrA1 protein, bands of approximately 100 and 150 kDa, presumably dimeric and trimeric HtrA1 complexes respectively, were also detected in high-serum CM in the absence of β-ME (Fig. 1A). Adding the reducing agent to CM samples greatly reduced the abundance of high molecular weight HtrA1 complexes after SDS-PAGE, suggesting that the oligomers are cross-linked by disulphide bridges. When probed by native PAGE, extracellular HtrA1 formed more complex high molecular weight conformations under high-serum than low-serum conditions (Fig. 1B).

To determine whether exogenously introduced HtrA1 also forms oligomeric complexes, we generated a human *HtrA1* construct tagged with an HA epitope at the N-terminus and V5/6xHis epitopes at the C-terminus (Fig. 1C). This construct was stably transfected into HEK293 cells (hereafter referred to as HEK-HtrA1 cells), which lack endogenous HtrA1 expression (Fig. 1D). Both anti-HtrA1 and anti-V5 antibodies revealed strong ∼50, ∼100, and ∼150 kDa bands in HEK-HtrA1 CM, similar to the complexes of endogenous HtrA1 secreted by HeLa cells. The C-terminal 6xHis tag also allowed purification of recombinant HtrA1 protein from HEK-HtrA1 CM using Ni-NTA columns (Fig. 1E). High molecular weight HtrA1 complexes were consistently recovered in high-serum HEK-HtrA1 CM, while serum-free culture only produced monomeric HtrA1. HtrA1 protein was also detected in the flow-through (unbound) fraction due to saturation of the Ni-NTA column.

These results suggest that secreted HtrA1 protein of both endogenous and exogenous origins can form disulphide bond-linked oligomer complexes, depending on cell culture conditions.

### Extracellular HtrA1 oligomerization can be promoted by small molecules

**Figure 2:**
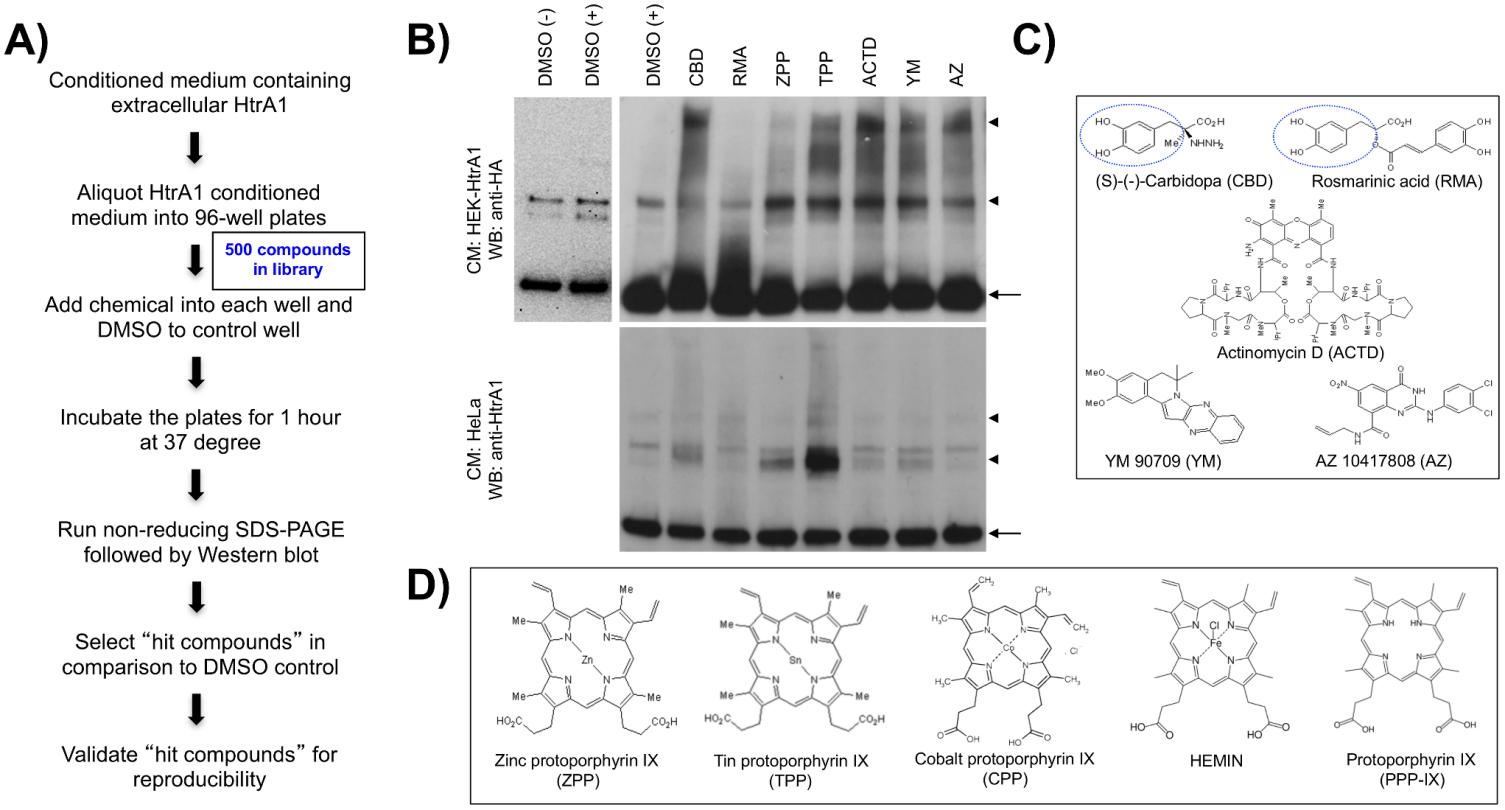
The formation of HtrA1 extracellular complexes can be affected by small molecules. (**A**) A schematic of the chemical intervention strategy for selecting molecules capable of affecting extracellular HtrA1 complex formation. “Hit compounds” were identified when the HtrA1 complex pattern was altered in comparison to DMSO treatment. (**B**) Conditioned serum from HEK-HtrA1 and HeLa cells was probed with anti-HA and anti-HtrA1 antibody respectively after treatment with the seven hit compounds. All compounds increased HtrA1 complex abundance (arrowheads) compared to the DMSO treated control. DMSO treatment did not alter complex formation. CBD: (S)-(-)-Carbidopa, RMA: Rosmarinic acid, ZPP: zinc protoporphyrin IX, TPP: tin protoporphyrin IX, ACTD: Actinomycin D, YM: YM 90709, AZ: AZ 10417808. (**C**) Schematic diagrams of the chemical structure of the non-MPP hit compounds. Dashed lines indicate a conserved chemical moiety in Carbidopa and Rosmarinic acid. (**D**) Schematic diagram of the chemical structures of the protoporphyrin IX-based metalloporphyrins used.

Based on the ability to detect oligomers of secreted HtrA1 protein in cell culture, we hypothesized that chemical intervention could modulate complex formation. To test this hypothesis, we screened a chemical library of 500 compounds for the ability to modulate extracellular HtrA1 oligomer formation. This library, including naturally occurring and synthetic compounds, was previously used for similar screens [27]–[30], [32]. In our screen, the HtrA1-containing CM from HEK-HtrA1 cells was subjected to incubation with individual chemicals from the library at 37°C degree for 1 hr, followed by non-reducing SDS-PAGE and immunoblot analysis. Of the 500 tested chemicals, we identified seven positive hits that consistently altered the formation of HtrA1 complexes, when compared to DMSO-treated CM in both HEK-HtrA1 and HeLa cells (Fig. 2A, B).

All seven positive hits increased the abundance of high molecular-weight HtrA1 complexes. Two compounds – (S)-(-)-carbidopa (CBD), an inhibitor of aromatic L-amino acid decarboxylase, and rosmarinic acid (RMA), a natural antioxidant – share a chemical moiety (dashed circles in Fig. 2C). The other unrelated compounds include actinomycin D (ACTD, a DNA-binding antibiotic), YM90709 (YM, an interleukin-5 receptor agonist) and AZ10417808 (an inhibitor of caspase-3). Noticeably, two positive hits belong to the metalloporphyrin (MPP) family, namely zinc and tin protoporphyrin IX (ZPP and TPP) (Fig. 2D). These two compounds are particularly intriguing because they are derived from protoporphyrin IX (PPP-IX), a biological intermediate in the heme biosynthetic pathway that accumulates in Erythropoietic protoporphyria (EPP) [1]. Thus, we focused our investigation on the MPP-HtrA1 interactions in the present study.

### Metalloporphyrins can directly interact with HtrA1 to enhance protease function

**Figure 3:**
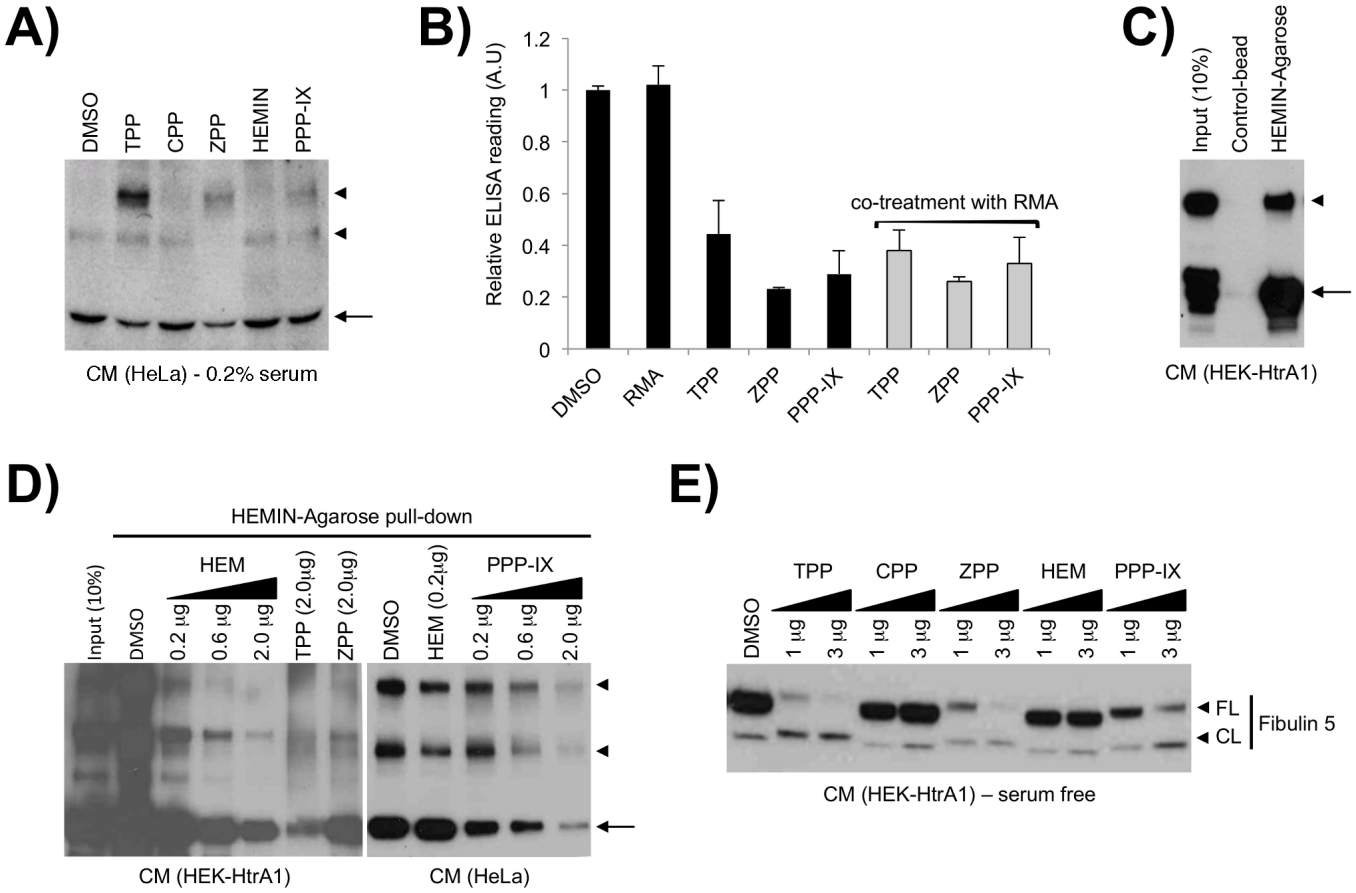
MPPs and HtrA1 appear to interact directly. (**A**) MPPs displayed differential activity in affecting The pattern of extracellular HtrA1 complexes (arrowheads) from HeLa cell low-serum conditioned medium is differentially altered by treatment with MPPs (25 mM, 37°C, 1 hr), as demonstrated when probed with polyclonal anti-HtrA1 antibody. (**B**) MPPs may induce conformational changes upon binding to HtrA1. The accessibility of N-terminal and C-terminal epitopes in the presence of MPPs was determined by ELISA and compared to DMSO controls. Error bars indicate the standard deviation. (**C**) Extracellular HtrA1 (arrows) could be precipitated from HEK-HtrA1 conditioned medium using HEMIN-conjugated agarose beads but not control, unconjugated agarose beads. (**D**) Competitive binding experiments using conditioned medium from HEK-HtrA1 or HeLa cells pre-incubated with MPPs revealed reduced recovery of HtrA1 (arrows) in the presence of competitor compounds when probed with polyclonal anti-HtrA1 antibody. (**E**) Degradation of Fibulin 5 in fixed HeLa cells treated with HtrA1 conditioned medium was enhanced in the presence of TPP, ZPP and PPP-IX. RMA: Rosmarinic acid, TPP: tin protoporphyrin IX, ZPP: zinc protoporphyrin IX, PPP-IX: protoporphyrin IX, HEM: HEMIN, CPP: cobalt protoporphyrin IX, CM: conditioned medium.

First, we postulated that additional MPPs not represented in the chemical library may also promote HtrA1 oligomer formation, and examined the effects of three additional MPPs; cobalt protoporphyrin IX (CPP), HEMIN (protoporphyrin IX conjugated to a ferric and chloride ion), and protoporphyrin IX (PPP-IX) (Fig. 2D). The conditions of MPP treatment were chosen to mimic the conditions of the original screen, with one exception: the serum concentration was reduced to 0.2% because bovine serum albumin (BSA) is known to interact with MPPs and ameliorate their effects [33]–[37]. Considering the high concentration of albumin in FBS, we used low-serum medium to reduce the interference with MPP-mediated HtrA1 complex formation. Indeed, these MPPs also promoted HtrA1 complex formation, with the greatest effects exerted by TPP, ZPP, and PPP-IX (Fig. 3A).

Next, we hypothesized that interaction with MPPs affects the presentation of specific epitopes on HtrA1. To test this possibility we purified monomeric HA-HtrA1-V5/6xHis protein from serum-free HEK-HtrA1 CM using a Ni-NTA column, and incubated it with three individual MPPs (TPP, ZPP, PPP-IX) with and without RMA. The resultant HA-HtrA1-V5/6xHis protein was captured by anti-V5 antibody on coated plate and detected with anti-HA antibody. Since the same amount of HA-HtrA1-V5/6xHis protein was used in all conditions, the ELISA signal reflected the relative accessibility of the V5 and HA epitope in monomeric (DMSO) or oligomeric HtrA1 (after incubation with MPPs and/or RMA). We found that incubation of purified HA-HtrA1-V5/6xHis protein with TPP, ZPP or PPP-IX greatly reduced the ELISA signal by 60–80%, when compared to the DMSO control (Fig. 3B). In contrast, RMA did not affect the ELISA signal, which correlates with its lesser ability to promote ∼150 kDa trimeric HtrA1 complex formation (Fig. 2B). These results suggest that interaction with MPPs changes the conformation of HtrA1 protein and the accessibility of its N- and C-terminal epitopes.

We then tested whether HtrA1 directly interacts with MPP compounds in a competitive manner. We found that HEMIN-conjugated, but not free agarose beads, pulled down both monomeric and oligomeric forms of HtrA1 in support of a direct physical interaction (Fig. 3C) [38], [39]. Moreover, the amount of HtrA1 protein recovered in HEMIN agarose affinity chromatography was diminished by pre-incubation of CM with TPP, ZPP, or PPP-IX in a dose-dependent manner, suggesting competitive binding to HtrA1 among MPPs (Fig. 3D).

Since MPPs promote the formation of oligomeric HtrA1 complex, which is associated with increased HtrA1 protease activity, we tested whether interactions with MPPs also up-regulates its ability to degrade Fibulin-5, a biological substrate of HtrA1 [3], [4]. We initially performed siRNA-mediated knockdown of *HtrA1* in HeLa cells to investigate the effect of MPPs on HtrA1 protease activity. While Fibulin-5 cleavage was reduced upon *HtrA1* knockdown, the effect of drug treatment was unclear (data not shown). Lightly methanol-fixed HeLa cells were used as the source of extracellular matrix following previous reports [3], [4], and serum-free CM was collected from HEK-HtrA1 cells to ensure high levels of extracellular HtrA1 without the interference of other proteases and protease inhibitors in the FBS. After exposure to HtrA1-containing CM, cellular extracts were subjected to immunoblotting analysis to compare the intact (CL) and cleaved forms (CL) of Fibulin-5. Pre-exposure to TPP, ZPP and PPP-IX enhanced the ability of HtrA1-CM to degrade Fibulin-5 in a dose-dependent manner, while exposure to CPP and HEMIN lacked this effect (Fig. 3E). Similar results were obtained using mouse retinal pigment epithelium/choroid explants as the substrate (data not shown).

Together, these results suggest that selective MPPs, specifically TPP, ZPP, and PPP-IX, interact with HtrA1 to promote oligomer formation and protease activity.

### CARASIL-associated mutations of HtrA1 may diminish interaction with MPPs

**Figure 4:**
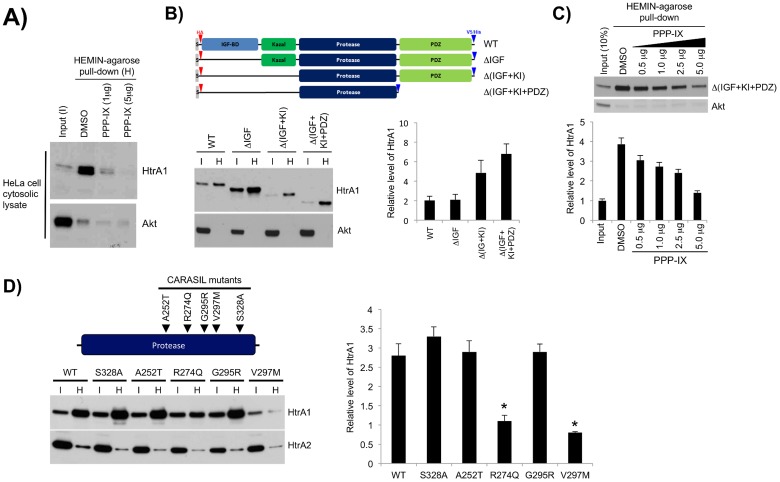
PPP-IX binds to the protease domain of HtrA1 and disease-associated mutations eliminate binding. (**A**) PPP-IX competed with HEMIN for endogenous HtrA1 binding. HeLa cell lysate was subjected to HEMIN pull down. Less HtrA1 was recovered in the presence of PPP-IX. Akt was used as a control and was not purified from the same pull down. (**B**) The HtrA1 protease domain was sufficient for interaction with MPPs. A schematic diagram of the deletion constructs tagged with HA and V5/hexa-His epitopes is shown (top). Cell lysates from HEK293 expressing variant HtrA1 proteins were subjected to HEMIN pull down and eluates (H) were examined for tagged protein with the anti-HA antibody (bottom left). The amount of recovered protein compared to input (I) is shown (bottom right). (**C**) PPP-IX competed with HEMIN for binding to the HtrA1 protease domain in a dose-dependent manner, with less HtrA1 protein recovery as PPP-IX concentration increased, revealed by immunoblotting for anti-HA. Akt was not similarly purified. The amount of recovered protein compared to input is shown (bottom). (**D**) Disease associated mutations reduced the interaction between HtrA1 and MPPs. The position of engineered single amino acid mutations in variant HtrA1 constructs is shown in the schematic diagram (top). Variant HtrA1 protein pulled down from transfected cell lysates with HEMIN agarose (H) was probed for HtrA1 (left panel) and the amount of recovered protein compared to input (I) was calculated (right panel). R274Q and V297 M significantly reduced the binding of HtrA1 to HEMIN (*: p<0.01). HtrA family member HtrA2 is not purified, demonstrating that the interaction is specific to HtrA1.

HtrA1 comprises four domains: an IGF-binding domain, a Kazal-type inhibitory domain, the protease domain and a PDZ domain, from the N- to C-terminus [2], [40]. To determine which domain is essential for MPP binding, we expressed truncated *HtrA1* constructs in HEK293 cells, and compared their capture by HEMIN-agarose beads in whole-cell lysates. While HtrA1 existed as monomers in whole-cell lysates, it interacted with HEMIN-agarose beads in a similar manner to extracellular oligomers (Fig. 4A).

We generated three *HtrA1* deletion constructs containing the HA and V5/6xHis tags. These are: ΔIGF, which lacks the IGF-binding domain; Δ(IGF+KI), which lacks the IGF-binding domain and the Kazal domain; Δ(IGF+KI+PDZ) devoid of the IGF-binding domain, the Kazal domain, and the PDZ domain, leaving only the proteolytic domain intact (Fig. 4B). All deletion constructs expressed protein that could be captured by HEMIN-agarose beads and detected by ant-HtrA1 antibody, suggesting that the protease domain is sufficient for MPP binding (Fig. 4B). Furthermore, despite a low expression level in input (I), truncated Δ(IGF+KI+PDZ) protein was more extensively enriched by HEMIN-agarose (H) mediated pull-down (Fig. 4B). The capture of Δ(IGF+KI+PDZ) by HEMIN-agarose was also diminished by PPP-IX in a dose-dependent manner, suggesting competition for the same binding site (Fig. 4C).

Because CARASIL-associated mutations of *HtrA1* are often found in the protease domain [16], [41], we tested whether these disease-related mutations impair the HtrA1-MPP interaction. We used site-directed mutagenesis to generate *HtrA1* constructs bearing one of five CARASIL-associated mutations: A252 T, R274Q, G295R, V297 M and S328A. Of these five mutations, R274Q and V297 M were found to negatively affect the purification of HtrA1 by HEMIN-agarose (Fig. 4D). These results suggest that selective CARASIL-associated mutations of *HtrA1* may interfere with its interaction with MPPs, though additional studies are needed to determine the consequence of this interference.

### HtrA1 is involved in PPP-IX induced apoptotic death

Our results thus far showed that MPP-HtrA1 interaction up-regulates HtrA1 activity and oligomer formation. Conversely, does the MPP-HtrA1 interaction facilitate the pro-apoptotic effects of MPPs, especially PPP-IX [26], [42]? Because ectopic expression of HtrA1 itself triggers cell death [21], we cannot examine this issue with a gain-of-function approach. Instead, we tested whether knockdown of *HtrA1* by siRNA attenuates PPP-IX-induced apoptosis in HeLa cells that express endogenous HtrA1.

**Figure 5:**
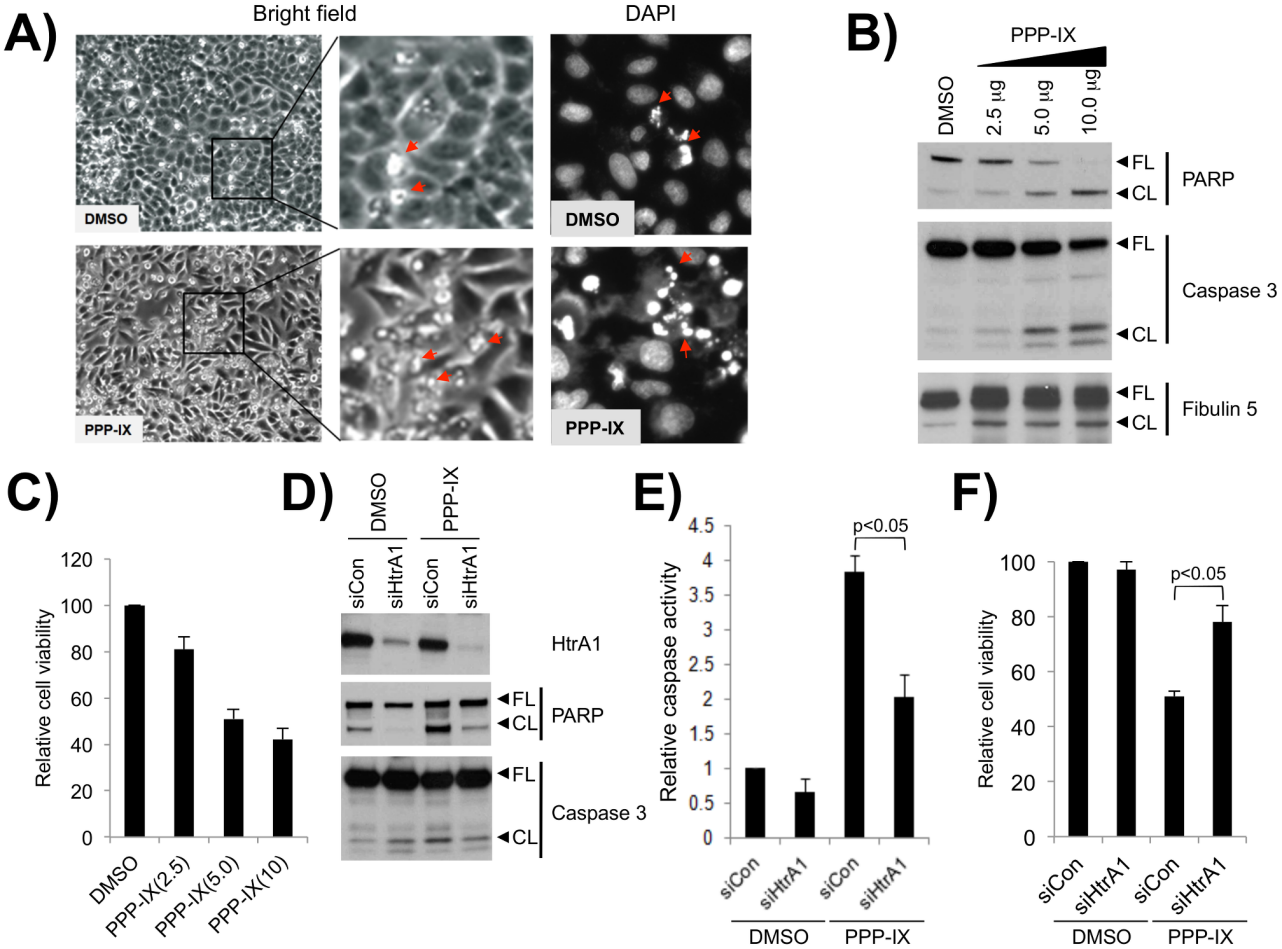
Loss of HtrA1 attenuates PPP-IX induced cell death. (**A–C**) PPP-IX treatment induced apoptotic cell death in HeLa cells. (**A**) Bright field images of HeLa cells treated with PPP-IX (lower left) revealed increased membrane blebbing (arrows) compared to DMSO treated cells (upper left). Dotted squares indicate the location of magnified views shown in the middle panel. DAPI staining of nuclei (right panel) revealed increased numbers of fragmented nuclei (arrows) in PPP-IX treated cells compared to DMSO treatment. (**B**) PARP and Caspase-3 are cleaved in lysates from HeLa cells undergoing cell death in response to increasing concentrations of PPP-IX. Fibulin-5 cleavage was used to confirm drug efficacy. (**C**) PPP-IX treatment reduced cell viability, as determined by the MTT assay, in a dose-dependent manner. (**D-F**) Knockdown of HtrA1 conferred resistance to PPP-IX induced cell death. (**D**) HtrA1 knockdown reduced the extent of PARP and Caspase-3 cleavage in PPP-IX treated cells. (**E**) The relative activity of Caspase-3/7 compared to that for DMSO treated, siCon transfected cells is shown. Caspase activation is reduced in siHtrA1 transfected HeLa cells undergoing PPP-IX induced cell death. (**F**) The viability of cells transfected with control (siCon) or HtrA1-targeting (siHtrA1) was assessed in the presence and absence of PPP-IX. PPP-IX-induced cell death was attenuated by loss of HtrA1. FL: full length intact protein, CL: cleaved protein.

When PPP-IX was added into HeLa cell culture, we observed membrane blebbing, condensation of the nucleic chromatin (Fig. 5A) and cell detachment from the plates, all indicative of cell death. These morphological changes were associated with decreased cell viability and dose-dependent cleavage of PARP and caspase-3 (Fig. 5B, C). Of note, increased cleaved Fibulin-5 was detected following the PPP-IX treatment, which suggests the induction of HtrA1 activity.

In contrast, in HeLa cells transfected with HtrA1-targeting siRNA (siHtrA1), PPP-IX-mediated cleavage of PARP and induction of caspase-3 activity were attenuated (Fig. 5D, E). The same effect was not observed in non-targeting control (siCon) transfected cells. Additionally, the viability of HeLa cells following PPP-IX treatment recovered from 53% in siCon-transfection to 78% after siHtrA-transfection (Fig. 5F).

Together, these results suggest that HtrA1 potentiates PPP-IX mediated cell death.

## Discussion and Conclusions

HtrA1 is a serine protease that is mostly secreted to degrade numerous extracellular matrix proteins, but it also exists within cells for some partially understood functions [6]–[7], [20]–[21]. Down-regulation of HtrA1 is associated with CARASIL, tumorigenesis, and increased resistance to chemotherapy, while HtrA1 hyperactivity is linked to arthritis and age-related macular degeneration [8]–[17]. Structurally, HtrA1 shares many characteristics of the HtrA family proteins, including oligomer conformation (homotrimers) and a C-terminal PDZ domain immediately after the trypsin-like protease domain [33]. However, unlike bacterial HtrAs, the mammalian HtrA1 is not regulated by PDZ domain-mediated allosteric activation, but rather through substrate-induced remodeling in the active site of its protease domain [6], [22]–[24]. This unique induced-fit mechanism raises the possibility that biological co-factors may interact with the protease domain of HtrA1 to promote its enzymatic activity and oligomerization.

We set out to test this hypothesis by screening a library of 500 chemical compounds for the ability to promote oligomerization of secreted HtrA1 protein in culture medium. Our screen identified seven positive hits, including two metalloporphyrins (MPPs) derived from the heme biosynthetic pathway. In extension experiments, we found that a subset of MPPs, including TPP, ZPP, and PPP-IX, share a strong ability to induce HtrA1 oligomerization. Interestingly, the ability to promote HtrA1 oligomers among MPPs correlates with the induction of its protease activity, as shown by the cleavage of Fibulin-5 (compare Fig. 3A & E). These results suggest that the MPP-mediated HtrA1 oligomerization is associated with an increased proteolytic activity. This is in agreement with previous studies on HtrA1 and bacterial DegP showing that higher molecular weight complexes can increase their protease activity [9], [10]. Of note, our use of Fibulin-5 instead of the generic fluorophore-conjugated β-casein substrate to measure the HtrA1 protease activity has two advantages. First, Fibulin-5 is a specific substrate for HtrA1 in vivo [18], [19]. Second, this system avoids interference of the inherent fluorescence of MPPs with the quantification of fluorophore-conjugated substrates.

One scenario to account for the oligomerization-activation coupling, in view of the induced-fit model, is that MPPs may directly interact with HtrA1 in the protease domain to induce conformational change [6], [22]–[24]. Consistent with this hypothesis, we showed that binding to MPPs alters the accessibility to epitopes in the N- and C-terminus of HtrA1 in an ELISA (Fig. 3B). We also showed that secreted HtrA1 protein can be pulled down by HEMIN-agarose and dissociated by TPP, ZPP, and PPP-IX, likely due to a higher binding affinity (Fig. 3D). Furthermore, truncation studies showed that the protease domain of HtrA1 is sufficient to interact with HEMIN-agarose beads (Fig. 4B), while a subset of CARASIL-associated mutations in the protease domain diminish this physical binding (Fig. 4D). Considering the protease domain is also sufficient to allow trimerization, these results support the notion that MPPs may directly interact with the protease domain of HtrA1 to promote its activity and oligomerization. It is interesting that HEMIN and CPP could interact with HtrA1 but exerted limited effects on HtrA1 oligomerization and activity, and suggests some level of selectivity in MPP effect. Future studies are warranted to compare the crystal structure of HtrA1 in the presence or absence of MPPs to further test this hypothesis.

Finally, the observed HtrA1-MPP interactions provide fresh insights into the mechanisms of protoporphyria and photodynamic therapy for cancer [1]–[4]. Both Erythropoietic protoporphyria (EPP) and the application of photosensitizers during photodynamic therapy result in PPP-IX and MPP-derivatives accruing in the body which, upon light exposure, emit reactive oxygen species that damage the connective tissue and skin or directly enter cells to induce apoptosis [5], [35]. Our results suggest that PPP-IX and MPPs may activate HtrA1 as part of their mechanism to damage cells and the connective tissue. For example, MPPs promote the HtrA1-medaited degradation of Fibulin-5 (Fig. 3E), while knockdown of HtrA1 attenuates PPP-IX-induced caspase-3 activity and cell death (Fig. 5D–F). Consistent with this notion, past studies have shown that overexpression of HtrA1 enhances chemotherapy-induced apoptosis [16]. Collectively, these findings suggest that induced HtrA1 activity may contribute to the cytotoxicity of PPP-IX and porphyrin-based photosensitizers.

Interestingly, preliminary data suggests that at least some of this effect might be dependent on intracellular HtrA1: treating cells with PPP-IX promotes intracellular HtrA1 complex formation and leads to reduced EGF and IGF signaling, pathways with which HtrA1 is known to interact (data not shown). This effect was also observed under serum rich conditions, despite the high availability of growth factors. Further studies into how HtrA1 and PPP-IX interact to modulate cell survival will be very informative, as the interplay between HtrA1 and porphyrin toxicity may have two important applications. First, past studies have uncovered several lead compounds for HtrA1 inhibitors [21]. It is conceivable that such small molecule HtrA1 inhibitors could be formulated as lotion to reduce photosensitivity and skin lesions in patients with EPP. Secondly, local ectopic expression of HtrA1 may enhance photodynamic therapy for cancer.

In conclusion, the present study suggests for the first time direct HtrA1-MPP interactions. While additional research is needed to scrutinize the molecular details and functional significance, our findings offer a new direction to elucidate the roles of HtrA1 in human diseases.
